# Superimposed high-frequency jet ventilation combined with continuous positive airway pressure/assisted spontaneous breathing improves oxygenation in patients with H1N1-associated ARDS

**DOI:** 10.1186/2110-5820-2-7

**Published:** 2012-03-06

**Authors:** Tobias M Bingold, Bertram Scheller, Timo Wolf, Jens Meier, Alexander Koch, Kai Zacharowski, Peter Rosenberger, Thomas Iber

**Affiliations:** 1 Clinic of Anaesthesia, Intensive Care Medicine and Pain Therapy, University Hospital Frankfurt am Main, Theodor-Stern-Kai 7, 60590 Frankfurt am Main, Germany; 2 Medical Clinic II, Infectious Disease, University Hospital Frankfurt am Main, Germany

## Abstract

**Background:**

Numerous cases of swine-origin 2009 H1N1 influenza A virus (H1N1)-associated acute respiratory distress syndrome (ARDS) bridged by extracorporeal membrane oxygenation (ECMO) therapy have been reported; however, complication rates are high. We present our experience with H1N1-associated ARDS and successful bridging of lung function using superimposed high-frequency jet ventilation (SHFJV) in combination with continuous positive airway pressure/assisted spontaneous breathing (CPAP/ASB).

**Methods:**

We admitted five patients with H1N1 infection and ARDS to our intensive care unit. Although all patients required pure oxygen and controlled ventilation, oxygenation was insufficient. We applied SHFJV/CPAP/ASB to improve oxygenation.

**Results:**

Initial PaO_2_/FiO_2 _ratio prior SHFJV was 58-79 mmHg. In all patients, successful oxygenation was achieved by SHFJV (PaO_2_/FiO_2 _ratio 105-306 mmHg within 24 h). Spontaneous breathing was set during first hours after admission. SHFJV could be stopped after 39, 40, 72, 100, or 240 h. Concomitant pulmonary herpes simplex virus (HSV) infection was observed in all patients. Two patients were successfully discharged. The other three patients relapsed and died within 7 weeks mainly due to combined HSV infection and in two cases reoccurring H1N1 infection.

**Conclusions:**

SHFJV represents an alternative to bridge lung function successfully and improve oxygenation in the critically ill.

## Background

The swine-origin 2009 H1N1 influenza A virus has become the predominant influenza virus worldwide since its identification. H1N1 influenza might cause acute respiratory distress syndrome (ARDS) and potentially result in extracorporeal membrane oxygenation (ECMO) therapy [1]. Experience from Australia and New Zealand describes an incidence of mechanical ventilation in 64.6% of H1N1 patients and 11.6% ECMO (with a mortality of approximately 21%) treated within the intensive care unit (ICU) [2].

Mortality rates of ARDS patients suffering from hypoxemia and/or hypercapnia remains high [3]. In this situation, ECMO therapy represents the standard of bridging lung function [2]. However, ECMO therapy is associated with unfavorable complications and high cost. In this report, we suggest an alternative strategy to bridge lung function: the use of superimposed high-frequency jet ventilation (SHFJV) in combination with continuous positive airway pressure/assisted spontaneous breathing (CPAP/ASB). This alternative ventilation strategy is based on jet ventilation to improve oxygenation at lower plateau pressure combined with assisted spontaneous breathing by CPAP/ASB to improve CO_2 _removal.

## Methods

In winter 2009-2010, five patients with ARDS (H1N1) were admitted to our ICU due to inadequate oxygenation by conventional mechanical ventilation from outside hospitals. ARDS was defined according to the definition of the American-European Consensus Conference on ARDS [4].

### Microbiology

Virological and microbiological tests were performed in bronchioalveolar lavage under sterile conditions. Influenza A H1N1 infection was determined by real-time reverse transcriptase-polymerase chain reaction (RT-PCR) assay. In addition, herpes simplex virus (HSV)-DNA, cytomegalovirus (CMV)-DNA, Epstein-Barr virus (EBV)-DNA, RSV, and adenovirus were tested by TAQMAN PCR. Atypical species, such as Mycoplasma, *Pneumocystis jiroveci*, or Legionella were screened through PCR. BAL, swabs, and blood cultures were taken routinely on admission and depending on clinical need.

### Mechanical ventilation

Patients were ventilated with a Monsoon I or Monsoon III jet ventilator (Acutronic Medical Systems, Hirzel, Switzerland, distributed by IfM GmbH, Wettenberg, Germany) combined with a standard respirator, Evita XL (Draeger, Lübeck, Germany) or Hamilton G5 (Hamilton Medical, Bonaduz/Switzerland, distributed by Heinen & Löwenstein GmbH, Bad Ems, Germany).

### Weaning protocol

• Spontaneous breathing within 12 h after admission

• Positive end-expiratory pressure (PEEP) primary 12-15 mbar

• Ppeak (peak pressure) < 30 mbar

• Pmean < 20 mbar

• FiO_2_, work pressure, and frequency of jet, ASB in adaption to blood gas analyses; goal: oxygen delivery (DO_2_) normal, lactate normal, pH 7.3-7.45

• Supine position 135° up to 12-14 h as long as oxygenation benefits

• ECMO (QUADROX PLS and ROTAFLOW RF 32, MAQUET GmbH &Co. KG, Rastatt, Germany) *Interventional Lung Assist *(ILA Membranventilator^®^, Novalung GmbH, Talheim, Germany) when CO_2 _> 50 mmHg, pH < 7.3 and no progress by conservative ventilation and sedation management

### Sedation

To achieve a RASS (Richmond agitation and sedation scale) of -3 to -4 propofol or midazolam, in two cases additionally with γ-hydroxybutanoic acid was used in combination with remifentanil or sufentanil and clonidine.

### Antiviral medication

All patients received oseltamivir, two patients in combination with ribavirin and amantadine up to 9 days. HSV was treated as standard with acyclovir; two patients received foscarnet-sodium when CMV coinfection was suspected or proven.

## Results

Five patients with severe influenza A H1N1 infection and refractory hypoxemia were ventilated between 2 and 16 days before admission. The transfer of these patients to our ICU was due to failure to improve during conventional ventilation (PEEP 10-15 mbar, pressure-controlled ventilation with P_peak _up to 35 mbar, FiO_2 _1.0).

On admission, patients presented with a paO_2_/FiO_2 _ratio of 58 to 79 (Murray score > 3.0; Table 1). After institution of SHFJV, oxygenation improved in all patients within 24 h (paO_2_/FiO_2 _ratio 105-306) with a PEEP of 8 to 15 mbar, P_peak _< 30 mbar, and P_mean _15-18 mbar. Therefore, the institution of SHFJV allowed improved oxygenation with adequate DO_2 _and a simultaneous reduction of lung injury, inducing high peak and mean pressures at the same time. Spontaneous breathing was set during 8 h after admission. Three patients required jet ventilation for up to 72 h. One patient required prolonged jet ventilation due to concomitant mycoplasma pneumonia, and the other due to persistent H1N1 pneumonia.

**Table 1:** Demographic data of patients, ICU admission data, and arterial blood gases with start of jet ventilation

**Patient no**.	1	2	3	4	5
**Age [yr]**	38	42	18	25	57
**Gender**	f	m	m	m	m
**Chronic disease/condition**	Obesity	Obesity	--	Ulcerative colitis	Obesity
**Scores at admission**					
**SOFA**	9	15	10	14	29
**SAPS II**	29	66	22	30	10
**APACHE II**	27	26	26	23	26
**Murray score**	3.75	3.75	3.25	3.25	3.5
**Laboratory findings at admission**					
**LDH [U/l]**	2245	708	1644	525	1348
**CK [U/l]**	2440	24	4335	58	534
**CRP [mg/dl]**	40.67	37.9	2.12	16.06	29.9
**PCT [ng/ml]**	2.5	0.9	4.1	1.2	0.7
**Leukocytes [/nl]**	4.19	12.55	7.79	42.4	2.77
**Arterial blood gases at admission**					
**pH**	7.358	7.270	7.352	7.40	7.33
**p_a_O_2 _[mmHg]**	79.3	79.1	68.7	58	75.6
**p_a_CO_2 _[mmHg]**	48.2	66.0	45.8	55	54.6
**F_i_O_2_**	1.0	1.0	1.0	1.0	1.0
**Lactate [mg/dl]**	19	29	10	97	10
**Arterial blood gases within 24 h after start of SHFJV**					
**pH**	7.356	7,539	7.291	7.30	7.343
**p_a_O_2 _[mmHg]**	113	122	188	105	133
**p_a_CO_2 _[mmHg]**	55.7	38,0	49.7	70.1	47,5
**F_i_O_2_**	0.8	0.4	1.0	1.0	0.8
**Lactate [mg/dl]**	14	31	9.0	80	12

SOFA = sequential organ failure score; SAPS II = Simplified Acute Physiology Score II; APACHE II = Acute Physiology and Chronic Health Evaluation II; LDH = lactate dehydrogenase; CK = creatinine kinase; CRP = C-reactive protein; p_a_O_2 _= arterial oxygen fraction; p_a_CO_2 _= arterial carbon dioxide fraction; F_i_O_2 _= oxygen fraction.

FiO_2 _was reduced from initially 1.0 to 0.4-0.6 in four cases. However, one patient with a ventilation period of 14 days before admission due to H1N1 and concomitant *Pneumocystis jiroveci *infection needed a FiO_2 _of 0.9 up to day 7.

Jet ventilation was performed in three cases with a work pressure of 1.2-1.7 bar, a frequency of 140-200/min (Monsoon I Jet ventilator); in two patients a novel jet ventilator was used and the work pressure was set to 0.5-0.9 bar and the frequency adjusted to 500-600/min (Monsoon III Jet ventilator).

**Figure 1:**
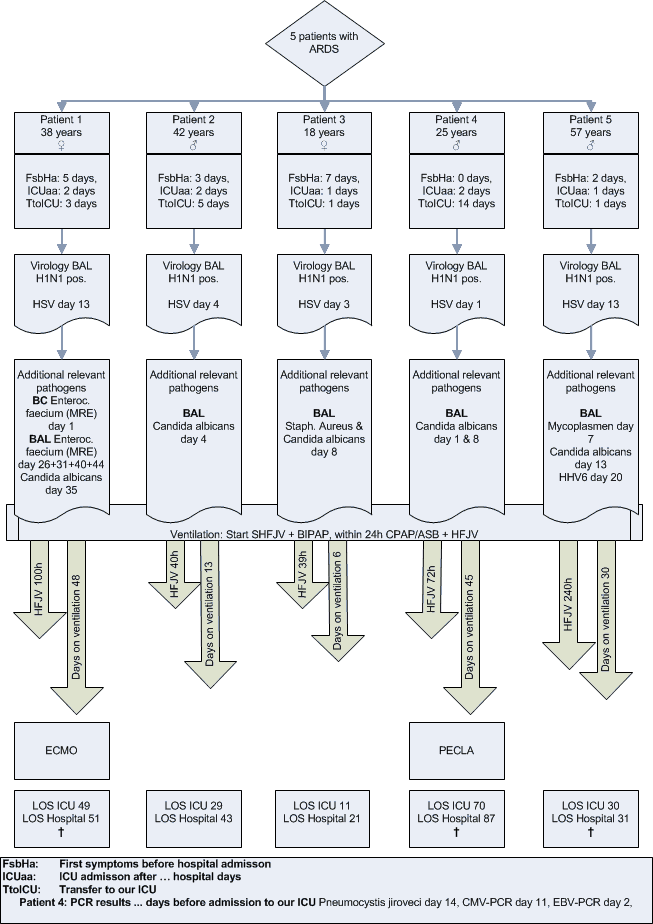
**Detailed patient data of ventilator treatment and viral infection**.

When only H1N1 infection was present, the oxygenation deficit was the leading clinical symptom. In cases with prolonged treatment (reinfection or concomitant infection with HSV, Mycoplasma) hypercapnia also was notable. Therefore, one patient received ECMO and one patient ILA therapy (Figure 1). The patient with ECMO therapy demonstrated improved oxygenation and normocapnia but deceased due to intracranial bleeding. This patient had histologic confirmed HSV pneumonia for 4 weeks without improvement after acyclovir/foscarnet treatment. A similar clinical pathway was observed in the two other patients who died.

Despite pulmonary failure, organ dysfunction was moderate. Some patients showed liver dysfunction; two of three nonsurvivors had acute renal failure. Vasoactive drugs were needed initially in all patients.

From anamnesis, four patients were obese; one patient had pre-existing medical complications (acute CMV colitis and acute *Pneumocystis jiroveci *pneumonia by ulcerative colitis). None of the patients had pre-existing immunodeficiency, such as HIV.

## Discussion

During ARDS, ECMO is seen as the standard therapy to improve oxygenation. The CESAR study demonstrated that in highly specialized centers, ECMO therapy may improve long-term outcome. However, this study had limitations within the study design, because the intervention and control group were treated at different centers [5]. In a review of 62 cases with ECMO therapy, the survival rate was 55% [6]; 17.8% of patients died as a result of ECMO-related complications, translated into a mortality of 8% due to ECMO alone. A retrospective U.S. database analysis (1986-2006) demonstrated a mortality rate of 50% in patients treated with ECMO. Although technical improvement of ECMO equipment translated into fewer cases of circuit rupture, renal insufficiency, pulmonary hemorrhage, inotropic medications, hyperglycemia, extremes of pH, arrhythmias, or hypertension became more common [7].

The primary goal for ARDS patients is to preserve an acceptable gas exchange without further injury to the lungs. High-frequency jet ventilation (HFJV) and high-frequency oscillatory ventilation (HFOV) are characterized by rapid delivery of small tidal volumes (V_t_) (1-3 ml/kg predicted body weight) at high frequencies (2.5-5 Hz). As such, P_mean _during jet ventilation is comparable to the pressure level of PEEP in conventional mechanical ventilation due to its minimal V_t_. Yet, P_peak _and P_mean _are markedly reduced with a reduction of tidal hyperinflation and tidal decruitment [8]. Furthermore, oxygen toxicity may be reduced as lower FiO_2 _often are required compared with mandatory ventilation [8]. This beneficial effect could be demonstrated in animal models of HFOV. In addition, pulmonary compliance improved, the infiltration of polymorph nuclear leukocytes was reduced, and tumor necrosis factor-α levels attenuated. As a result, injury to alveoli and membranous bronchioles was reduced [9].

HFOV mainly differs from HFJV by active in- and expiration, whereas HFJV only uses active inspiration and passive expiration. Thus, HFJV allows patients spontaneous breathing, preserving muscles of diaphragm and intercostal musculature. This is a major advantage, because early spontaneous breathing significantly reduces deleterious organ-organ interactions, e.g., lung-liver interactions, and improves liver perfusion [10].

One major problem of jet ventilation is CO_2 _clearance due to very low V_t_. There are two options in resolving this problem. The simplest approach is to establish spontaneous breathing. However, patients might be sedated and therefore need superimposed CPAP/ASB to improve tidal inflation until sedation is adapted. In the case of severe hypercapnia, the use of Interventional Lung Assist (ILA) can improve CO_2 _clearance.

We observed a high mortality rate, which can be -explained, in part, by patient selection. All of our patients presented in this report were admitted with fixed hypoxemia despite optimized conventional ventilation. Two patients presented a prolonged period of hypoxemia and mechanical ventilation before admission. One of these patients was already on mechanical ventilation 14 days due to septic CMV-colitis and *Pneumocystis jiroveci *pneumonia with poor prognosis. The other patient was admitted to our ICU after a prolonged period of severe hypoxemia and septic shock with hypoperfusion. All five patients had a HSV-PCR-positive BAL, confirmed via lung biopsy in one patient. Viral coinfection with HSV may cause and prolongs a status of persistent immunosuppression during severe H1N1 infection, which is associated with a high risk of death. This hypothesis is supported by findings of Monsalvo and colleagues, who found pathogenic immune complexes as previously unknown biological mechanism for the unusual age distribution of severe H1N1 infections [11].

## Conclusions

Although jet ventilation is actually not in the focus of ARDS treatment, we were able to demonstrate that SHFJV represents an alternative of lung-protective ventilation during ARDS. Compared with ECMO, it is easier to use, associated with fewer complications, cost-effective, and can be used in secondary and tertiary centers. Therefore, SHFJV is an alternative approach to improve lung function and oxygenation in patients suffering from ARDS. Nevertheless, this report is limited by the small study size, including a heterogeneous patient collective. Therefore, a controlled study with SHFJV to treat patients with ARDS is required for an evidence-based conclusion.

## Competing interests

This study was performed at the University Hospital Frankfurt am Main and was internally funded. It is independent of any pharmaceutical interest and has no potential conflict of interest.

## Authors' contributions

All authors participated in study design. TB, TI, BS, AK, JM, and KZ participated in data collection. TB, TI, BS, JM, AK, PR, and KZ analyzed and interpreted the data. TB, TI, PR, and KZ drafted the report. TB, TI, TW, PR, and KZ critically reviewed the report. All authors read and approved the final manuscript.
